# Multiple Hazard Uncertainty Visualization Challenges and Paths Forward

**DOI:** 10.3389/fpsyg.2021.579207

**Published:** 2021-07-19

**Authors:** Lace Padilla, Sarah Dryhurst, Helia Hosseinpour, Andrew Kruczkiewicz

**Affiliations:** ^1^Spatial Perception, Applied Cognition and Education (SPACE) Lab, Cognitive and Information Sciences, University of California Merced, Merced, CA, United States; ^2^Department of Pure Mathematics and Mathematical Statistics, Winton Centre for Risk and Evidence Communication, University of Cambridge, Cambridge, United Kingdom; ^3^International Research Institute for Climate and Society, The Earth Institute, Columbia University, Palisades, NY, United States; ^4^Red Cross Red Crescent Climate Centre, The Hague, Netherlands

**Keywords:** compound risks, hazards, uncertainty, visualization, multiple, cognitive effort, trust, communication

## Abstract

Making decisions with uncertainty is challenging for the general public, policymakers, and even highly trained scientists. Nevertheless, when faced with the need to respond to a potential hazard, people must make high-risk decisions with uncertainty. In some cases, people have to consider multiple hazards with various types of uncertainties. Multiple hazards can be interconnected by location, time, and/or environmental systems, and the hazards may interact, producing complex relationships among their associated uncertainties. The interaction between multiple hazards and their uncertainties can have nonlinear effects, where the resultant risk and uncertainty are greater than the sum of the risk and uncertainty associated with individual hazards. Effectively communicating the uncertainties related to such complicated systems should be a high priority because the frequency and variability of multiple hazard events due to climate change continue to increase. However, the communication of multiple hazard uncertainties and their interactions remains largely unexplored. The lack of practical guidance on conveying multiple hazard uncertainties is likely due in part to the field’s vast expanse, making it challenging to identify entry points. Here, we offer a perspective on three critical challenges related to uncertainty communication across various multiple hazard contexts to galvanize the research community. We advocate for systematic considerations of multiple hazard uncertainty communication that focus on trade-offs between complexity and factors, including mental effort, trust, and usability.

## Introduction

In early April of 1991, Mount Pinatubo in the Philippines began producing precursory activity to a volcanic eruption (Self et al., 1993). Scientists from the Philippines and the United States quickly established monitoring stations around Mount Pinatubo and accurately forecasted the eruption on July 15, 1991. Officials evacuated the areas around Mount Pinatubo 48h before the eruption, saving thousands of lives (Westby and Phillips, 2016). At the same time that Mount Pinatubo was producing 17 megatons of sulfur dioxide and ash, typhoon Yunya also made landfall in the Philippines (Self et al., 1993; Gill and Malamud, 2014). The typhoon’s massive rains combined with large amounts of ash caused mudflows and collapsed roofs, resulting in unanticipated casualties of people who had been evacuated to unfortified structures. This paper is motivated by the question: Can the visualization community develop methods to convey multiple hazards and their uncertainties in a way that would help policymakers and the public prepare for such hazard interactions and reduce their vulnerability to hazards? In the case of Mount Pinatubo, what challenges could have been overcome to visualize a volcanic eruption combined with a typhoon and the associated uncertainties that would have helped people take appropriate action when the risk was elevated with both hazards occurring concurrently?

**Figure 1:**
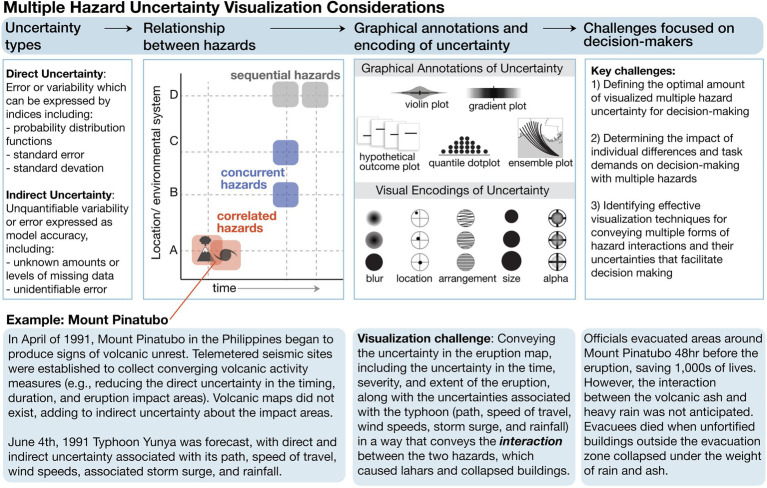
Overview of multiple hazard uncertainty visualization considerations discussed in this paper. Uncertainty types based on discussions in Padilla et al. (2021). Relationship between hazards based on Balch et al. (2020). Summary of graphical annotations redrawn from Padilla et al. (2021). Mount Pinatubo example based on accounts in Gill and Malamud (2014).

Quantifying the interactions of risks and uncertainties for multiple hazards is an active research area (for reviews, see Balch et al., 2020; Raška et al., 2020). As illustrated in Figure 1 (see the relationship among hazards), multiple hazards can occur in the same location and at the same time (i.e., *correlated hazards*), as in the 1991 volcanic eruption and typhoon in the Philippines. The correlation of the eruption and typhoon at Mount Pinatubo produced a compound risk, where the risk created by the interaction of the two hazards was greater than the sum of each hazard (Gill and Malamud, 2014). *Concurrent hazards* occur simultaneously but in different locations, such as when the August Complex fire in 2020 burned over a million acres in northern California at the same time the SQF Lightning Complex fire burned hundreds of thousands in the southern part of California (CALFire, 2020; for an alternative definition, see De Luca, 2020). *Sequential hazards* occur at the same location but at different times (Balch et al., 2020; for a discussion of terminology, see Cutter, 2018). For example, in 2010, Haiti experienced a magnitude 7.0 earthquake that killed 250,000 people, followed by a cholera epidemic, and then a category two hurricane (PAHO/WHO, 2011). Sequences of hazards can have a nonlinear additive effect on vulnerability, where the resultant risk is greater than the sum of the risk associated with individual hazards (Kappes et al., 2012; Haqiqi et al., 2021; Kruczkiewicz et al., 2021).

Even though researchers are making significant advances in quantifying the interactions between multiple hazards (e.g., hazards that are meaningfully interconnected by space, time, and/or environmental systems), visualizing the relationship between multiple hazards with uncertainty remains largely unexplored. A recent review found that of 693 studies that examined multiple hazards, only 5% visualized the hazards using vulnerability maps, and no studies visualized uncertainties (Raška et al., 2020). The underrepresentation of visualizations of multiple hazards and their uncertainties is surprising since a substantial body of work demonstrates that visualizing uncertainty can significantly improve decision quality (for review, see Padilla et al., 2021).

Communicating the uncertainty associated with multiple hazards is imperative to facilitate individuals’ informed decision-making on an institutional scale (e.g., national-level anticipatory action plans) and on a more personal basis (e.g., evacuating when there is a perceived risk from a hazard; Morss et al., 2016). For example, the humanitarian sector is increasingly developing standard operating procedures for anticipatory action (Pichon, 2019), including impact-based risk assessments that require policy-makers to understand both geophysical and socioeconomic uncertainty (WMO, 2015; Forzieri et al., 2016; Taylor et al., 2021). Decision-makers without an understanding of the uncertainty in a forecast may be underinformed, placing undue levels of confidence in a forecast (Fischhoff, 2012; Fischhoff and Davis, 2014).

Further, entities and researchers suggest that when groups communicate uncertainty, they can demonstrate the trustworthiness of their science by showing a commitment to transparency (e.g., O’Neill, 2012; Stocker et al., 2013). Indeed, prior research suggests that communicating uncertainty can maintain perceptions of trustworthiness (Van Der Bles et al., 2020). Given the pragmatic and ethical reasons why communicating uncertainty is critical, organizations, including the Intergovernmental Panel on Climate Change (IPCC), advocate for communicating uncertainty about natural events (Stocker et al., 2013).

To motivate the visualization community to address the gap in research on visualizing multiple hazards with uncertainty, we offer a discussion of three critical multiple hazard communication challenges for policy and personal decision-making. We also discuss potential paths forward for research on visualizing uncertainty in multiple hazards. In this paper, we focus our discussion on challenges for uncertainty visualizations of multiple hazards for policy decisions, such as governmental disaster-risk reduction and personal choices. Efforts are also needed to advance uncertainty visualization of multiple hazards for scientific data analysis and education but are beyond this discussion’s scope.

## Multiple Hazard Uncertainty Communication Challenges and Paths Forward

Multiple hazard scenarios have several forms of uncertainties associated with each hazard, which could be visualized for decision-makers. Researchers have made efforts to classify these forms of uncertainty within various typologies (e.g., Van Asselt and Rotmans, 2002; Walker et al., 2003; Morgan, 2009; Spiegelhalter, 2017; van der Bles et al., 2019).

As demonstrated in Figure 1 (see uncertainty types), visualization researchers commonly classify uncertainties into two groups: *direct* and *indirect* (Padilla et al., 2020b). Researchers can characterize direct quantified uncertainties (i.e., epistemic; Spiegelhalter, 2017) with mathematical expressions denoting error or variability in measurements. For example, in the case of Mount Pinatubo, uncertainties existed within the target period of the eruption forecast, along with the extent of the impact. Additionally, the typhoon forecast had multiple forms of direct uncertainty, including the uncertainty associated with the path of the storm, speed of forward motion, wind speeds, central pressure, storm surge, and rainfall. For Mount Pinatubo/Typhoon Yunya, at least nine forecast parameters and uncertainties could be visualized along with their interrelations.

*Indirect* uncertainty (i.e., ontological; Spiegelhalter, 2017) is also associated with each forecast parameter, which is uncertainty that cannot be quantified directly, such as unknown amounts or levels of missing data, or unidentifiable error that enters the modeling pipeline (Pang et al., 1997). For example, forecasts for Mount Pinatubo eruptions did not previously have impact maps, and the maps were quickly generated (Westby and Phillips, 2016). The team of scientists from the Philippines and the United States had limited time to create and test the impact maps, thereby leading to unquantifiable amounts of error. Forecasters can express this type of indirect uncertainty as subjective expert interpretations of forecast or model accuracy (Budescu et al., 2012; IPCC, 2014).

The eruption of Mount Pinatubo and Typhoon Yunya had at least nine forecast elements, each with both direct and indirect uncertainties (nine elements * direct and indirect = 18 uncertainties) and 18 possible interactions (18 interactions * direct and indirect uncertainty = 36 uncertainties). If forecasters were to fully communicate all the forecast parameters and uncertainty, they would need to visualize 54 types of uncertainties. Presenting policy-makers or the general public with such complex information (i.e., data with numerous components and nonlinear relationships) could overburden their decision making. However, what is the “right” number of uncertainties to visualize, and for whom? Should some uncertainties be combined before the visualization step to make composite risk indices (if so, which ones?), or should some uncertainties be left out? Can decision-makers deal with more uncertainty before a forecasted hazard, such as when developing preparatory action plans?

### Defining the Optimal Amount of Visualized Multiple Hazard Uncertainty for Different Decision-Makers

The first challenge we highlight is the need for research to define the optimal amount of visualized uncertainty to support decision-making by policy-makers and the general public. We argue that hazard communicators should consider trade-offs when deciding how to visualize the uncertainty associated with multiple hazards and their interactions (Politi et al., 2011; Chen et al., 2016). There may be a *sweet spot* where decision-makers are provided with sufficient uncertainty information to increase decision efficacy but not so much that their decisions become overcomplicated, leading to their reliance more on heuristics than on the data (Sullivan-Wiley and Gianotti, 2017). For example, in the case of Mount Pinatubo/Typhoon Yunya, rather than visualize all 54 types of uncertainty, forecasters could summarize the indirect uncertainties across each hazard into one account of model accuracy. Alternatively, forecasters could integrate some forecast components, such as volcanic impact area and storm surge, into an estimate of vulnerability and visualize the aggregated uncertainty. Research is needed to provide guidance about the ideal amount of uncertainty, which hazard communicators could use to decide when uncertainty aggregation or reduction is needed.

Further, researchers have found that making decisions with uncertainty requires more of our limited *mental effort* than judgments without uncertainty (Sprenger and Dougherty, 2006). Researchers in psychology and cognitive science have established that mental effort involves the controlled use of our limited ability to process information at a given time (Trujillo, 2019). As the number of uncertainties in a multiple hazard visualization increases, decision-makers will be forced to expend more of their limited mental effort until they reach their maximum. When decision-makers’ mental effort becomes overloaded, they will not be able to process additional uncertainty information, which is highly likely in disaster/crisis response and preparedness situations and could produce adverse effects on disaster management decision-making (Comes et al., 2013).

*Intelligent transparency*, which inspires our recommendations for future directions, focuses on demonstrating the trustworthiness of science to help viewers more accurately evaluate if the information is deserving of trust (Gustafson and Rice, 2020). As O’Neill (2012) asserts, “Our aim – everybody’s aim – is surely to trust the trustworthy, but not the untrustworthy.” We advocate for research that aims to provide people with a useful amount of uncertainty to understand the possible outcomes of multiple hazard interactions and evaluate if the science is honest, competent, and reliable. At the same time, these visualizations should not overburden decision-makers with superfluous uncertainty information that harms their decision-making to the point where they discount and or discredit the available uncertainty information. Work is also needed to define the applicability of such trade-offs (e.g., sweet spot) to various multiple hazard situations. For example, would the sweet spot be the same for a multiple hazard scenario with a volcanic eruption and a hurricane compared to a heatwave and a flash flood?

### Determine the Impact of Individual Differences and Task Demands on Decision-Making With Multiple Hazards

When examining the optimal amount of uncertainty to convey, researchers should consider that different viewers may have varying abilities to incorporate visualized uncertainties into their decision-making processes. Experienced multiple hazard risk decision-makers may view multi-hazard risk as a system, and they may benefit from seeing unaggregated uncertainties since they can easily build up a holistic understanding of the interacting risk in their minds. In contrast, the general public may try to understand the risk associated with each hazard (within that system) and then face the daunting task of fusing the uncertainties together in a piecemeal fashion (Tilloy et al., 2019). Further, decision-makers vary in the amount of mental effort they have available to consider multiple forms of visualized uncertainties and their interactions (Padilla et al., 2019). Future research should examine how mental effort limits interaction with various hazards and tasks, including decisions related to disaster-risk reduction within high-stress environments in the humanitarian sector.

In humanitarian and disaster contexts, tension is also present between the possible increase in mental effort and time needed to process visualizations with uncertainty and the lack of time to prepare for many hazards – both geophysical and nongeophysical (Zhang et al., 2002; Richardson et al., 2010; Comes, 2016). In such moments, people may rely on their automatic, and sometimes biased, judgments of uncertainty visualizations rather than systematically evaluating the multiple uncertainties presented to them (Padilla et al., 2018).

Future research also needs to account for emotions and prior experiences, and how specific hazards are weighted based on affect (e.g., a volcano eruption may take cognitive precedence because it may be perceived as more threatening than rain). Uncertainty provokes anxiety (Greis et al., 2017), and some decision-makers may become uncomfortable with depictions of uncertainty (Hullman, 2019). If processing uncertainty of a single hazard already provokes anxiety, being presented with multiple uncertainties and their interactions could increase anxiety considerably, leading to situations where decision-makers are biased toward more risk-averse actions (Lauriola et al., 2007; Fiala, 2017; Bourdeau-Brien and Kryzanowski, 2020). Further, decision-makers may not realize the degree to which the risk-aversion is leading them to underestimate the genuine risk. As stress uses mental effort (Qin et al., 2009), people will likely have less mental effort available to consider multiple hazard uncertainties during stressful hazard events. The negative impacts of stress may play a more significant role for traditionally underserved populations (Evans and Schamberg, 2009), commonly the intended beneficiaries of disaster risk reduction strategies, but governance of these populations may be lacking (Kruczkiewicz et al., 2018).

Trust is a crucial predictor of attention to scientific experts (e.g., Bleich et al., 2007) and the likelihood of taking preparatory action (Losee and Joslyn, 2018). A large body of research finds that trust is influenced by prior experiences with hazard forecasts (e.g., Wall et al., 2017), particularly after an anticipated hazard did not occur (i.e., false alarms; Dow and Cutter, 1998; Savelli and Joslyn, 2012; LeClerc and Joslyn, 2015). High rates of false alarms lead to poor decision-making and decreased trust (LeClerc and Joslyn, 2015). However, less work has examined how multiple hazard uncertainties impact trust among the public and high-stakes end-users. For example, we do not know how the increased possibility of false alarms will impact viewers’ trust in situations where multiple hazards are forecast or how previous experiences may influence perceptions of trust.

Two fundamental components of an individual’s judgment about who and what to trust include apparent intent and competence (Fiske et al., 2007; Fiske and Dupree, 2014), which can be demonstrated by communicating uncertainty (Jensen, 2008). Whereas communicating uncertainty signals trustworthiness in institutions by demonstrating a commitment to transparency and scientific credibility (e.g., O’Neill, 2012, 2018), giving too detailed an account of multiple uncertainties could lead people to view the research as incompetent, and then to switch to other less complicated but less reliable sources.

In line with the intelligent transparency approach (O’Neill, 2018), we advocate for research seeking to find the sweet spot in multiple hazard communication to foster trust but not provide superfluous information. Research is needed to examine how to optimize the amount of uncertainty visualized in multiple hazard scenarios for a wide range of decision-makers and task demands, particularly for decision-makers who might experience high levels of anxiety, time pressure, stress, and diminished trust.

### Identifying Effective Visualization Techniques for Conveying Multiple Forms of Hazard Interactions and Their Uncertainties That Facilitate Decision Making

**Figure 2:**
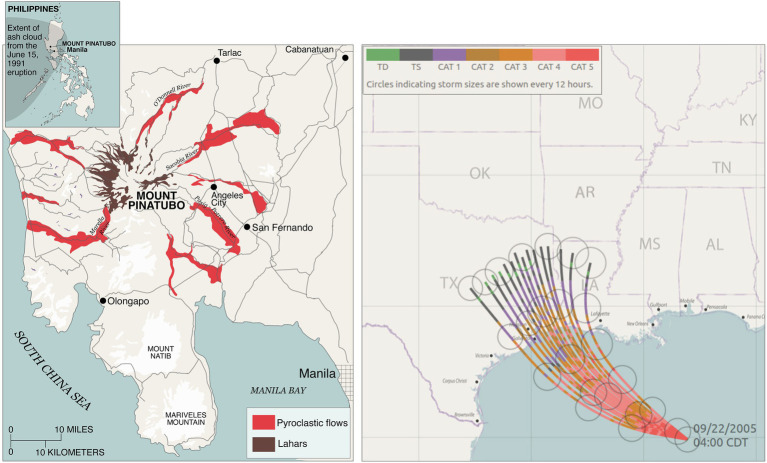
Left, map of Mount Pinatubo pyroclastic flows, lahars, and ash impact areas (redrawn from https://pubs.usgs.gov/fs/1997/fs113-97/). Right, an example of an ensemble hurricane forecast map showing the path, size, and intensity of the forecasted storm along with the associated uncertainties (reprinted with permission from Liu et al., 2019).

Reasoning with visualizations of common forms of probability is challenging for both novices and experts (Belia et al., 2005). For example, hurricane path visualizations are one of the most studied hazard visualizations, and all currently available visualization techniques produce some misunderstanding about a storm’s path (Cox et al., 2013; Ruginski et al., 2016; Padilla et al., 2017, 2018, 2020a). A few studies have examined how to communicate various types of components of one hazard, such as hurricane path, storm size, and intensity (see Figure 2; Liu et al., 2019). However, no work has systematically studied best practices for visualizing uncertainty related to two (or more) hazards and their interactions, including the uncertainty that results from the co-occurrence of two hazards.

Future work is needed to examine how graphical annotations and visual encodings of uncertainty can be used to convey uncertainties’ interactions (see Figure 1 for examples of empirically tested graphical annotations and visual encodings of uncertainty). As previously discussed, multiple hazards may interact differently (as shown in Figure 1, the relationship between hazards). Research is needed to identify uncertainty visualization techniques that are effective for each type of multihazard interaction. For example, Figure 2 shows a redrawn eruption impact map produced by United States Geological Survey for Mount Pinatubo (Newhall, 1997) and an empirically validated ensemble hurricane forecast technique that shows the uncertainty in the storm’s path, size, and intensity (Liu et al., 2019). Research is needed to determine how to combine the information from both visualizations to convey interacting risk in situations similar to Mount Pinatubo erupting at the same time and location as Typhoon Yunya making landfall.

In addition to visualization design guidelines, such as minimizing clutter, research should consider the appropriate amounts of uncertainty to display differences in decision-makers’ abilities to use visualized uncertainties, prior experiences, and trust in the forecasts.

## Conclusion

With the rise in the frequency and severity of natural-hazard-driven disasters (Field et al., 2012), effective communication of the uncertainty associated with multiple hazards is increasingly essential. This paper demonstrates the need for future work in multiple hazard uncertainty communication and highlights barriers to visualizing multiple hazards and their uncertainties for policymakers and the public. We highlight the concept of intelligent transparency (O’Neill, 2012) as a guiding principle in uncertainty communication that can help researchers consider the trade-off in visualization complexity, decision-maker abilities, and trust. A future set of priorities in this space should be reviewed and refined by the transdisciplinary actors within it – using the challenges shared here as a starting point to convene and facilitate a discussion and critical reflection. Such research would require a large-scale interdisciplinary effort, incorporating knowledge from visualization design, disaster preparedness, climate science, policy, and governance.

## Author Contributions

LP led the writing of this article along with making intellectual contributions to this work. SD contributed both to the writing and development of concepts and arguments. HH added valuable insights about the writing and injected clarity into each section. AK incorporated expertise in disaster relief management and added structure and conceptual development to the writing. All authors contributed to the article and approved the submitted version.

### Conflict of Interest

SD was formerly employed by Frontiers.

The remaining authors declare that the research was conducted in the absence of any commercial or financial relationships that could be construed as a potential conflict of interest.
